# The Medical Healing of Souls: a strategy for welcoming post-pandemic mental health

**DOI:** 10.1590/0034-7167-2022-0331

**Published:** 2023-12-04

**Authors:** Angelica Yolanda Bueno Bejarano Vale de Medeiros, Eliane Ramos Pereira, Rose Mary Costa Rosa Andrade Silva

**Affiliations:** I Universidade Federal Fluminense. Niterói, Rio de Janeiro, Brazil

**Keywords:** Logotherapy, Health Care Professional, Pandemics, User Embracement, Mental Health, Logoterapia, Profesional de la Salud, Pandemias, Acogimiento, Salud Mental, Logoterapia, Profissional da Saúde, Pandemias, Acolhimento, Saúde Mental

## Abstract

**Objective:**

to reflect on the applicability of the Medical Healing of Souls (MHS) by health professionals as a welcoming strategy in post-pandemic mental health.

**Methods:**

a theoretical and reflective study, based on Viktor Frankl’s philosophy, based on the book “The Doctor and the Soul, From Psychotherapy to Logotherapy” and scientific literature.

**Results:**

the study was structured in two discursive approaches: MHS in the field of health; The applicability of MHS in post-pandemic mental health care.

**Final considerations:**

MHS can be used in mental health care, in health emergencies, promoting a more humane performance of health professionals, facilitating the integration of inevitable suffering into a meaningful life.

## RESUMO

**Objetivo:**

refletir sobre a aplicabilidade da Cura Médica de Almas (CMA) por profissionais de saúde como estratégia de acolhimento em saúde mental pós-pandemia.

**Métodos:**

estudo teórico e reflexivo, fundamentado na filosofia de Viktor Frankl, baseado no livro “*The Doctor and the Soul, From Psychotherapy to Logotherapy*” e literatura científica.

**Resultados:**

o estudo foi estruturado em dois enfoques discursivos: A CMA no campo da saúde; A aplicabilidade da CMA no acolhimento em saúde mental pós-pandemia.

**Considerações finais:**

a CMA pode ser usada na atenção em saúde mental, em emergências sanitárias, promovendo uma atuação do profissional de saúde mais humanizada, facilitando a integração do sofrimento inevitável em uma vida com sentido.

## RESUMEN

**Objetivo:**

reflexionar sobre la aplicabilidad de la Cura Médica de Almas (CMA) por parte de los profesionales de la salud como estrategia de acogida en la salud mental pospandemia.

**Métodos:**

estudio teórico y reflexivo, basado en la filosofía de Viktor Frankl, a partir del libro “The Doctor and the Soul, From Psychotherapy to Logotherapy” y literatura científica.

**Resultados:**

el estudio se estructuró en dos enfoques discursivos: CMA en el campo de la salud; La aplicabilidad de la CMA en la atención de la salud mental pospandemia.

**Consideraciones finales:**

la CMA puede ser utilizada en la atención a la salud mental, en emergencias de salud, promoviendo una actuación más humana del profesional de la salud, facilitando la integración del sufrimiento inevitable a una vida con sentido.

## INTRODUCTION

The COVID-19 pandemic has had an unprecedented impact on public health worldwide. Over the past 50 years, the World Health Organization (WHO) has recorded more than 6,940,000 deaths since the beginning until the first half of 2023^(1)^. The impact of the pandemic can be divided into four waves: the first addresses the burden on health systems in providing quick care to patients; the second is related to the reduction of resources in the health area for the care of other acute clinical conditions; the third is interruption in health care for various chronic diseases^(2)^; for instance, during the pandemic, mental care and suicide prevention services were interrupted^(3)^; and the fourth includes the increase in mental disorders directly caused by COVID-19 or its secondary consequences^(2)^. Among them, we can highlight the biological repercussions of the virus, uncertainties about the infection, the growing number of deaths, the death of close people, social isolation, financial impacts on families, interruption of treatments due to lack of access, among others^(2-3)^, which, as a consequence, have triggered a 25% increase in the prevalence of anxiety and depression worldwide in the first year of the pandemic. For this reason, the WHO has issued a wake-up call to all countries to intensify health services and support in mental health^(3)^.

The pandemic’s psychological impact has highlighted human fragility and vulnerability, to the point that health professionals are increasingly confronted with a new type of patient, a new class of neurosis, a new type of suffering, whose characteristic is the fact that it does not represent a disease in the proper sense of the term, but an existential frustration, an emptiness, an inability to attribute meaning to suffering, a common characteristic of people with depression and suicidal thoughts^(4)^.

In this perspective, psychiatrist, creator of logotherapy, Viktor Emil Frankl, proposed a way of acting for doctors of his time who, when faced with patients experiencing situations of suffering imposed by fate (natural disasters; epidemiologies; pandemics; the death of loved ones; the experience of a terminal illness; the pain of a chronic illness; the amputation of a physical limb; the loss of a motor ability; the impossibility of having a child; the birth of a child with congenital deformities; caring for a family member with a disability, among others), a sense of meaninglessness, or existential emptiness should perform the Medical Healing of Souls (MHS)^(4)^.

The term coined by Frankl is a proposal to humanize the performance of all health professionals so that they have a welcoming approach to the person who suffers, calling on their patients to have attitudes of seeking and finding the meaning of life in their inevitable suffering, aiming at transforming an adverse situation into a personal achievement^(5)^. It should not be confused with the function of the priest or the pastor, who preach the salvation of the soul, nor with psychotherapy, which has been concerned with men’s ability to work and enjoy life, but it is a medical ministry, a mission in medicine practice, “to train human beings in the face of their suffering”^(4)^. For a better understanding of the theme, below, there is a brief description of the origin of logotherapy and the MHS.

### Logotherapy: a theoretical approach to Viktor Frankl’s thought

Viktor E Frankl (1905-1997) was born in Vienna, Austria, in the cultural and artistic cradle of Europe and the birthplace of modern psychology. From a young age he was a scholar and follower of Sigmund Freud (psychoanalysis) and Alfred Adler (individual psychology), but, for not believing that human persons walk through the world under the force of impulses (principle of pleasure-psychoanalysis), and not agreeing that human beings were in the world in search of power and the conquest of superiority (principle of power-individual psychology), he developed his own theory called existential analysis, stating that the awareness of responsibility for one’s existence is the foundation of human existence and, consequently, the search for the meaning of life (will to meaning-logotherapy). That is to say that his theory intends to bring awareness to “freedom for” and “responsibility” in the face of the constraints of existence^(4)^.

Frankl’s theory was inspired by the philosophical approaches of phenomenology and existentialism, founding the psychotherapeutic school called logotherapy: therapy for the meaning of life, a proposal that was put to the test with his own experience, when he became a prisoner in concentration camps during the Second World War and, after his release, argues the direct relationship between the lack of meaning in life and psychological distress^(4-5)^, an experience described in his famous work called “Man’s search for meaning”, a worldwide bestseller (1985).

Frankl proposes a vision of the human being, recognizing them as a unit in the totality that includes body, psyche and spirit (*noos*). This spiritual dimension, exclusive to human beings, is the source of meaning of life, and includes not only religiosity, but all evaluative, intellectual and artistic expression^(4)^. Frankl states that this dimension does not fall ill, and, unlike the body and mind, it has resources to face adverse situations, such as self-transcendence and self-distancing. The first is related to meaning of life, the ability to always be directed towards something or someone, to have a goal, it is like a leap of faith, which refers to trust, believe, act and seek something greater than oneself, found in love, in the other and in God whom it serves^(4,6)^. Self-distancing, on the other hand, is considered a virtue of taking a distance from a situation or the posture one is assuming in relation to it, moving away from the somatic and psychic conditions and determinants to contemplate them from another perspective^(4,6)^.

**Figure 1:**
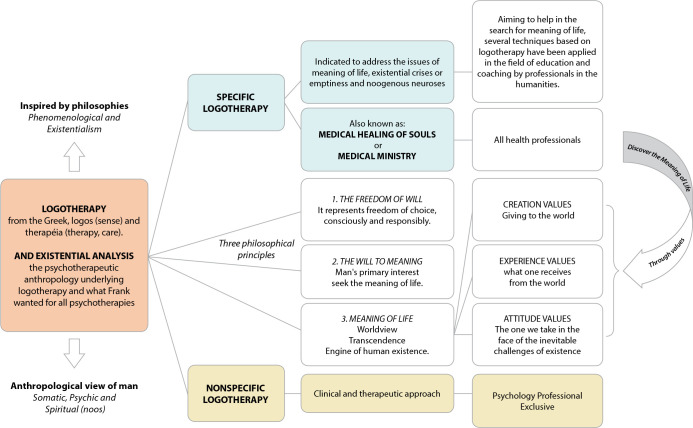
Comprehensive syntheses of Viktor Frankl’s theory

Logotherapy itself is more than psychotherapy, it is a philosophical anthropology applied to life, therefore, a praxiology constituted by praxis with the following characteristics: teleological: from the Greek, *τέλος*, purpose, and -*logía*, study, is the philosophical study of the ends, i.e., of the purpose, goal or aim, something to do (search for meaning); strategic: seeks to create bonds and relate to others; communicative: the possibility of doing everything with a logos, a meaning and a reason for being; integrative: connects knowledge with other knowledge, facing human problems in various fields; inclusive: includes the spiritual dimension and a person’s search for their meaning in life; interactive: dimensional relationships are interactive, not isolated behaviors. From a dimensional, dialogical perspective, it becomes a valid proposal today^(6)^. Figure 1 presents the main terms of Viktor Frankl’s theory.

Logotherapy and existential analysis is divided into specific and nonspecific logotherapy. The first is known in the health area as MHS or Medical Ministry, whose focus is on issues of existential emptiness defined by Frankl, as the massive neurosis of our time, an assertion that the being lacks meaning, characterized by a feeling of boredom, hopelessness, lack of interest, indifference, among others, which can cause psychosocial disorders, such as drug addiction, aggression, depression and suicide. This approach is intended for practice by health professionals^(4)^.

Non-specific logotherapy is focused on the clinical and psychotherapeutic process, an exclusive field of psychologists who can and/or should also work on questions about the existential void, i.e., perform a specific logotherapy in every psychotherapeutic process^(4,6)^. The three philosophical principles of logotherapy comprise freedom of will, will to meaning and meaning of life. The first dazzles the free and responsible human being, who not only reacts to stimuli or obeys impulses, but has the capacity to respond, therefore, it is conceived as being conscious of making decisions in the face of life’s constraints, with the freedom to submit or transcend the determinations^(4,6)^.

The will to meaning dazzles the human being as a self-transcending being capable of directing oneself with a purpose towards something or someone other than oneself, finding meaning^(4,6)^. Finally, meaning of life can be understood as the cognitive and affective perception of values (what is of greater importance and meaning), which gives direction, orientation, purpose and personal coherence^(6)^. Considered the engine for human existence, it is linked to a unique, unrepeatable situation and perceived when the human being realizes values, through the ability to love, work and endure suffering, also known as: experience values: first, it comprises everything that one receives from the world, in relationships, in love and in nature; creation values: includes everything that can be contributed to the world, when they create, work or donate; and attitude values: the posture of facing inevitable suffering, starting from the spiritual capacity of being responsible and making a favorable decision despite the inevitable^(4,6)^.

Frankl’s proposal about MHS is a doctor-patient intervention that suggests a welcome and follow-up as a “*medicus humanus*”: a doctor who not only treats as a doctor, but acts as a man to “*homo patiens*”, to the man who suffers, through the ability to see beyond the illness, to the being who feels and who, despite the suffering, still fights for a meaning^(4-6)^.

Considering the impact of the pandemic on mental health worldwide, this reflection suggests the possibility that other health professionals use MHS as a welcoming strategy. This study is also justified by the ongoing research of the authors at the Translational Qualitative Research Nucleus in Spirituality and Emotions in Health (Qualitees), registered in the directory of the Brazilian National Council for Scientific and Technological Development (CNPq - *Conselho Nacional de Desenvolvimento Científico e Tecnológico*) of the *Escola de Enfermagem Aurora de Alfonso Costa* at the *Universidade Federal Fluminense*, whose transdisciplinary focus advances in studies of human existence, affective, spiritual, psychosocial and well-being dimensions of people in the context of health/disease or risk situations, using logotherapy and meaning of life as a theoretical and methodological framework^(7)^, and due to the scarcity of literature on the subject in the current post-pandemic period, suggesting an opportunity for study and deepening.

## OBJECTIVE

To reflect on the applicability of MHS by all health professionals as a strategy for welcoming post-pandemic mental health.

## METHODS

This is a theoretical and reflective study, based on the philosophy of Viktor Frankl, based on the book “The Doctor and the Soul, From Psychotherapy to Logotherapy”, bibliographic collection of authors and national and international scientific literature. A non-systematic review of studies that dealt with the theme “MHS in mental health care in the context of the COVID-19 pandemic” was carried out. The search was carried out in December 2022. The databases consulted were: Virtual Health Library (VHL); Google Scholar; Online Scientific Electronic Library (SciELO); and Virtual Nursing Library (COFEN). Studies in any language, with no time restriction, and which correspond to the theme of this study were included. The terms used were: medical healing of souls, healing of souls, logotherapy, pandemic, COVID-19, mental health and reception, combined with each other through Boolean operators AND and OR. Studies of a religious nature or with proposals other than MHS were excluded. A reading of titles and abstracts of articles and other literary findings, such as guides and books, was carried out, conveniently selecting the most relevant ones that contributed to the discussion of this reflection.

## RESULTS

The discussion of the study was structured in two categories: *The Medical Healing of Souls in the field of health; The applicability of the Medical Healing of Souls in post-pandemic mental health care*.

### The Medical Healing of Souls in the field of health

Frankl’s anthropological thought rescued a person’s spirituality, seeking to humanize psychotherapy and the therapist-patient relationship. These paradigms illuminated Frankl’s life and his professional performance: “I wanted to be a doctor and I succeeded; I tried to be a good doctor and I hope I didn’t let anyone down; but, in the end, I decided to remain a man...” and “I’m not a psychiatrist to deal with men’s illnesses. I am a psychiatrist to care for patients’ humanity and the person’s spiritual”^(5)^.

MHS was born from the beginning of its investigations even before concentration camps. In the “love” for man and the “respect” for their humanity, Frankl proposes that the doctor-patient relationship involves “presence, attitude and invitation” to be with the other in their suffering, because there is still a question for this one: what else can you do?^(4)^. Health professionals’ attitude must go beyond the analysis of what caused their patient’s suffering and understand their suffering, but also summon them to face the challenges of destiny and to conceive, together with them, new alternatives for living that are full of meaning^(4)^.

In the field of health care, the founder of modern nursing, Florence Nightingale (1820-1910), draws attention to the fact that “the important thing is not what makes fate for us, but what we make of it”. Facing a health problem, it is not about removing the impact of the pain itself, but about reversing the perspective: instead of seeing the path in the context of the pain problem, starting to see the pain problem in the context of the meaning that gives dignity to life^(4,6)^.

Another of the most recognized nurses who adopted MHS was Joyce Travelbee (1926-1973), influenced by the Franklin current, which developed the theory of interpersonal relationship, proposing the importance of working on spirituality, meaning of life and love with patients^(7)^. For Travelbee, spiritual care in the area of nursing is oriented towards promoting a sense of life, which facilitates the integration of unfavorable contexts, such as pain and suffering, through the freedom and responsibility inherent in decision-making, which promotes control competence about the consequences^(7)^.

In turn, the Qualitees research group has been expanding its investigations using the theme “meaning of life” in nursing education, enabling undergraduate students to discover the meaning of nursing practice, aiming at a more humanized praxis^(7)^. It also conducts research aimed at revealing the meaning of life for patients in the specialties of cardiology and oncology.

In cardiology, a study carried out in the mental health nursing consultation at the outpatient clinic analyzed the spiritual dimension of 6 patients and verified the importance of finding a meaning in life for coping with the disease, making it possible to respond to unfavorable conditions in a mature and daring way, as the human spirit is rich and capable of overcoming any circumstance and thus resuming life^(8)^.

In the field of oncology, one of the ongoing studies is based on the proposal of psychiatrists Dr. William Breitbart and Dr. Greenstein, researchers at Memorial Sloan-Kettering Cancer Center, in New York City, who, in the year 2000, based on assumptions developed by Viktor Frankl, created a technique called “meaning-centered psychotherapy” for patients with cancer. They aim to study the demands of advanced cancer patients: the loss of spiritual well-being; the need to have meaning in life; existential suffering; and the presence of psychological co-morbidities such as depression, anxiety and stress^(9)^.

Encouraged by the importance of spiritual well-being, they aimed to help patients in their experience of attributing meaning to their lives, in addition to peace and purpose, even when close to the end of life. Currently, there is a growing interest in spiritual well-being, triggering a new wave of interventions aimed directly at this type of population^(9)^.

**Figure 2:**
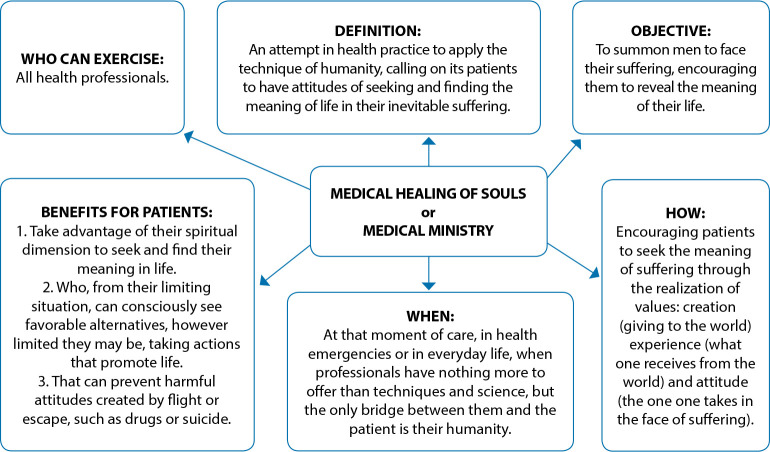
Comprehensive synthesis of the Medical Healing of Souls

For his part, Frankl cited several examples in which other specialties of medicine can use MHS: surgeons, who need to accommodate their patients when they are faced with an inoperable case, or when they must cripple patients for life, amputating a limb; so does dermatologists, who treat disfigured cases; general practitioners, who must treat disabled people, since in all cases life is more than the limb to be amputated, a good appearance, or the limitations that illnesses can impose on a person^(4)^. Figure 2 presents the synthesis of MHS.

Thus, MHS can be adopted by all health professionals, regardless of their specialty, to encourage the person who suffers to face their suffering condition with meaning, communicating hope^(4)^.

### The applicability of the Medical Healing of Souls in post-pandemic mental health care

The COVID-19 pandemic has triggered stressors of magnitudes never faced on a global scale^(2)^, with an increase in anxiety and depression due to the various socio-affective, economic, and health losses and numerous situations perceived as threatening by people during and after the pandemic^(3)^. Depression is related to Frankl’s concept of existential emptiness, a condition that may increasingly be taking many people to health services, with or without the presence of a physical condition of illness, with the expectation of finding answers to their existential questions through health professionals^(4-5)^.

Faced with this scenario, more and more patients are in need or, perhaps, it could be said, demanding a differentiated mental health care, attention that helps them to deal with a destiny that they can no longer shape, but that only can dominate and control, enduring and suffering with meaning.

From the COVID-19 pandemic, the need arose to create inclusive government policies that place mental health on the agenda of Primary Health Care, seeking bio-psycho-spiritual care for people in distress^(2)^. In turn, welcoming strategies in the first contact with the patient have been consolidating^(10)^. Below, we listed some of those that dialogue closely with MHS: encourage (without pressuring) people to talk about their situation, listening to them with care, respect and acceptance; help the person identify their needs and concerns, priorities and possibilities; encourage patients to name their feelings; recognize the patient as someone who can find an answer to their situation; see patients, even in a suffering condition, as a spiritual being both in their questions of faith and in their ability to find meaning in suffering; encourage the unveiling of meaning through values, love, for something or someone, the work or task to be performed, care for someone and the legacy to be built; help to reflect on the best alternatives to deal with the situation; reflect on what things give meaning to their life and for which it is worth continuing to live; contemplate the support network and ask for professional help, if necessary, to continue in this process of adaptation and overcoming to consolidate the meaning of life; and encourage to care for their spiritual needs^(2,4,10)^.

Thus, the post-pandemic health reception proposal is not only concerned with the body’s and mind’s health, but with the health of souls (thirst of emotions, feelings and affections, etc.). For Frankl, it is healthy when it remains what it intrinsically is, i.e., a being aware of their responsibility towards life and the response they must give to their suffering, in his words: “Because it will not be what life has to offer, but what each person has to give their own life, to the world, to others”^(4)^. Concluding this reflection, we emphasize that the impact of the pandemic has triggered existential questions, resulting in the need for humanized care so that both health professionals and patients can enjoy human potential, “their spirituality” of being able to attribute meaning to suffering, experiencing a sense of healing of souls.

## FINAL CONSIDERATIONS

The impact of the pandemic on mental health draws attention to humanized care, as more and more people seek health care in search of answers to challenges beyond the psychophysical dimension. Psychiatrist Viktor Frankl proposed a path of intervention for caring for a person’s humanity, the healing of souls, with the objective of helping them to face the inevitable suffering through revealing the meaning of life. With the intention of infusing comfort, faith and hope, health professionals encourage their patients to respond to suffering in a conscious way: freely and responsibly, with the certainty that even in suffering it is possible to find meaning. This awareness promotes the search for solutions and coping attitudes in favor of life, preventing fight-and-flight behaviors such as drugs and suicide. Several studies in the areas of nursing, cardiology and oncology have been using MHS in recent years as a health care strategy. It is concluded that MHS can be a humanized care strategy for all health professionals in mental health care in health emergencies, pandemics and post-pandemics, facilitating the integration of inevitable suffering into a meaningful life.
